# Analysis and Comparative Assessment of Basic Tribological Properties of Selected Polymer Composites †

**DOI:** 10.3390/ma13010075

**Published:** 2019-12-22

**Authors:** Jerzy Jozwik, Krzysztof Dziedzic, Marcin Barszcz, Mykhaylo Pashechko

**Affiliations:** 1 Department of Production Engineering, Faculty of Mechanical Engineering, Lublin University of Technology, Lublin 20-618, Poland; 2 Department of Computer Science, Electrical Engineering and Computer Science Faculty, Lublin University of Technology, Lublin 20-618, Poland; k.dziedzic@pollub.pl (K.D.); m.barszcz@pollub.pl (M.B.); 3 Department of Fundamentals of Technology, Fundamentals of Technology Faculty, Lublin University of Technology, Lublin 20-618, Poland; mpashechko@hotmail.com

**Keywords:** polymers composites, measurement, friction, sensors

## Abstract

Phenomena occurring in the contact area between two mating bodies are characterised by high complexity and variability. Comparisons are usually made between parameters such as the coefficient of friction, friction force, wear and temperature in relation to time and friction path. Their correct measurement enables the proper evaluation of tribological properties of materials used in the friction pair. This paper concerns the measurements of basic tribological parameters in the friction of selected polymer composites. Knowing the tribological properties of these composite materials, it will be possible to create proper operating conditions for kinematic friction pairs. This study investigated the coefficients of friction, friction force and temperatures of six polymer composites: cast polyamide PA6 G with oil, PA6 G with MoS_2_, polyoxymethylene POM with aluminium, polyethylene terephthalate PET with polytetrafluoroethylene PTFE, PTFE with bronze, and PTFE with graphite. The friction surface was also examined using an optical system and computer software for 3D measurements. As a result, PA6-G with oil was found to be the best choice as a composite material for thin sliding coatings.

## 1. Introduction

Friction is one of the most common phenomena in the world around us. It is also one of the main processes taking place between the mating elements of machines and devices. Friction processes are accompanied by wear and tear of friction pair elements, which makes the operational efficiency of these elements worse [1]. The tribological wear of materials is a result of abrasion, cracking and crushing of material particles, adhesion of the mating element surface, and tribo-chemical reactions occurring on the surface of friction, which is discussed in many works [2,3,4,5]. Materials used for friction pairs are subjected to tribological tests in order to determine their operational suitability. The complexity of processes occurring in the area of contact between mating elements makes it necessary to consider many different tribological parameters and mechanical properties of materials used in friction pairs. Many studies have attempted to mathematically model the contact area and simulate the occurring forces in order to better understand them. Waddad et al. have developed a multi-scale strategy for thermomechanical simulation of frictional systems such as brakes, taking into account the scale of the contact interface phenomena which is much lower than the macro scale of the system. They used the multi-scale strategy to study the thermomechanical behaviour of a pin-on-disc system. On a macro scale, they considered a finite element model to model the system components. On a micro scale, the thermal and mechanical contact problems were solved considering surface roughness and wear. They used semi analytic methods accelerated with the Fast Fourier Transform and optimisation techniques [6]. Pashechko et al. also used multi-criteria analysis to evaluate many tribological parameters and mechanical quantities, which allowed them to assess their influence on the wear resistance of Fe-Mn-C-B coatings alloyed with selected elements [7,8]. The most frequently measured and considered parameters in tribological studies are the coefficient of friction, the friction force, the degree of wear, and the temperature in the contact area. Tribological tests are carried out under laboratory conditions using tribotesters. Tribotesters very often differ in their designs and operation modes [9,10,11]. Sometimes, the material surface is also tested to ensure the accuracy of results, as knowing the nature of variation makes it easier to identify the type of wear and tear. Microscopic and spectroscopic examinations are conducted to evaluate microstructures and phase transitions [12,13,14,15].

Highly wear-resistant materials continue to be the subject of many research works. Prospective materials for sliding friction pairs include polymers and polymer composites. Polymer composites are characterised by the synergy of properties of every component. A characteristic feature of these materials, when compared to widely used metal alloys, is their relatively low weight combined with high resistance to corrosion and various substances. Therefore, polymers and polymer composites with the above properties are used in various industries such as automotive, aerospace, shipbuilding, and biomedical [16,17,18,19]. Various types of fibres and fillers, including matrix-reinforcing nanoparticles, are used in polymeric composites to significantly improve their properties including tribological characteristics [20,21]. For tribological applications, inorganic powders obtained by various methods such as sol-gel, impregnation, plasma impregnation or high energy grinding method are used. With an appropriate filler content, it is possible to create a composite material for specific tribological applications. Typically, the filler content is between 15% and 40% [22,23,24]. Polytetrafluoroethylene (PTFE) based composites with different fillers [25,26] are characterised by good tribological properties. Skoneczny et al. investigated the mechanical and tribological properties of polymeric composites based on polytetrafluoroethylene. They showed that the best mechanical properties were found in a PTFE composite with a 40% bronze content. On the other hand, the tribological tests showed that the lowest wear was observed for the polytetrafluoroethylene-based composite containing 25% bronze powder and 15% graphite and for the composite with a 25% carbon content [27]. Song at al. [28] also showed very good tribological properties of the PTFE composite reinforced with carbon fibres and graphite. Another group of polymers with good tribological properties are polyamides (PA). In their work, Pocznik et al. investigated the tribological properties of polyamide PA6 under dry friction conditions, at different velocities and load forces. The research showed that tribological properties are highly dependent on contact configuration. The tribological properties of a stationary steel pin sliding against a rotating polymer disc (SS/PA6) as well as of a self-mated PA6/PA6 contact were found to substantially depend on the contact conditions [29]. Gebretsadik et al. demonstrated that the PA66 composite containing 25% glass fibre in the sea water environment is characterised by good tribological properties. The lowest values of the coefficient of friction were obtained using a lubricant in the form of artificial seawater and solution of group II metal salts and NaHCO_3_. Significantly higher friction coefficients were observed under dry friction conditions [30]. Another polymeric material used in tribological applications is polyoxymethylene (POM). Mao et al. studied unreinforced polyoxymethylene and polyxymethylene reinforced with 28% glass fibre (GFR POM). The research was conducted on a specially designed gearing where the gears were made of the tested materials. They demonstrated that the life of GFR POM gears is higher than that of the gears made of POM alone [31]. Józwik et al. investigated the tribological properties of polymers and polymer composites such as PA6-G with oil, PA6-G with MoS_2_, POM with aluminium, PET with PTFE, PTFE with bronze or PTFE with graphite. The best tribological properties were observed for polyamide PA6-G with oil [32]. Practical applications for the tested materials in machine design and research were presented in the works [33,34,35,36,37,38,39,40,41,42,43,44].

## 2. Materials and Methods

The following materials were tested in this study: POM with aluminium, PET with PTFE, PTFE with bronze, and PTFE with graphite. Table 1 presents the tested materials and their basic properties such as density, filler content, thermal conductivity, and operating temperature range. 

**Figure 1:**
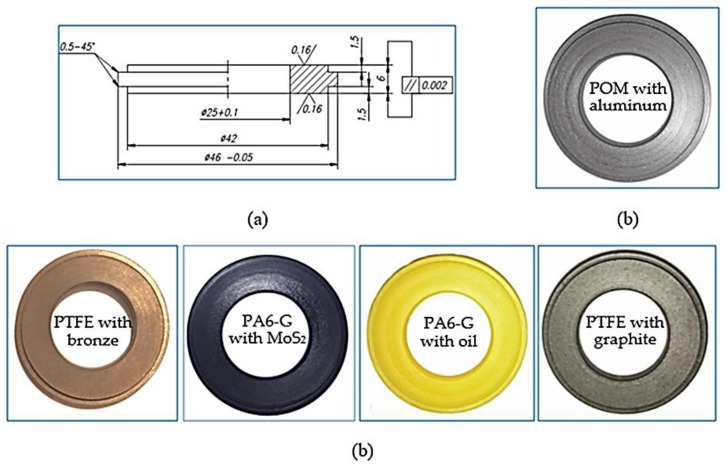
View of the samples used in the study: (**a**) sample dimensions, (**b**) specimen view.

**Figure 2:**
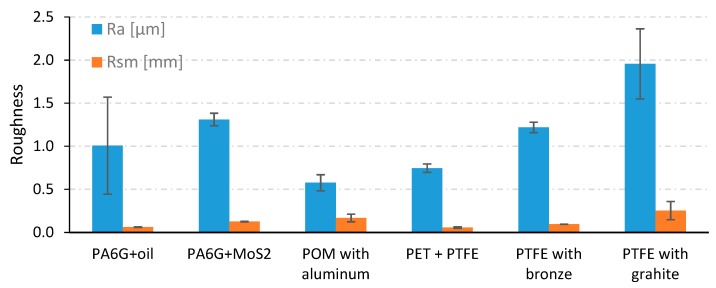
Roughness parameters Ra and Rsm.

According to the manufacturer, polyamide PA6-G with MoS_2_ or mineral oil has a high resistance to friction wear, tear strength and high dynamic loads. Therefore, it is used, among other things, in the manufacture of slats, gears and guides. POM is resistant to most chemical compounds, and the addition of aluminium improves its tribological properties. Therefore, it is most often used for sliding bearings and tension rollers in machines used in the food industry. It can operate at temperatures ranging from −30 °C to 105 °C. PET with the addition of PTFE is a modified PET polyester reinforced with PTFE fibres. This significantly improves its sliding properties and overall wear resistance. It is particularly recommended for sliding applications because it has very good properties in this regard, as well as high abrasion resistance and good mechanical strength. It can be used for direct food contact as it meets all the requirements for compliance with food safety regulations. PTFE with added bronze or graphite has very good tribological properties due to the fact that it is based on a PTFE polymer. It is characterised by a very low coefficient of friction and a very high resistance to abrasion. Graphite and bronze are characterized by faster heat dissipation, that is why their long-term operating temperature ranges from −200 °C to even +220 °C. The specimen materials were supplied in 1 m long solid bars with an external diameter of 45 mm. The sample bars were cut into 10 mm slices and machined by turning and grinding. In the middle of the external wall height, a 3 mm thick flange was made. Along the axis of symmetry of the samples, a 25 mm diameter hole was drilled through. Sanding paper of various grit sizes was used to grind the surface of the samples. Finally, the samples were sanded with abrasive paper of grain size P1000 (FEPA P standard), which corresponds to an average grain size of 18.3 µm. The abrasive treatment resulted in 6 mm thick samples, as shown in Figure 1. According to the standard, the surface roughness should be Ra = 0.16 µm. However, the actual surface roughness of the samples deviated slightly from the norm. It depended on the type of polymer composite. The initial roughness of samples after the preparatory work is shown in Figure 2.

**Figure 3:**
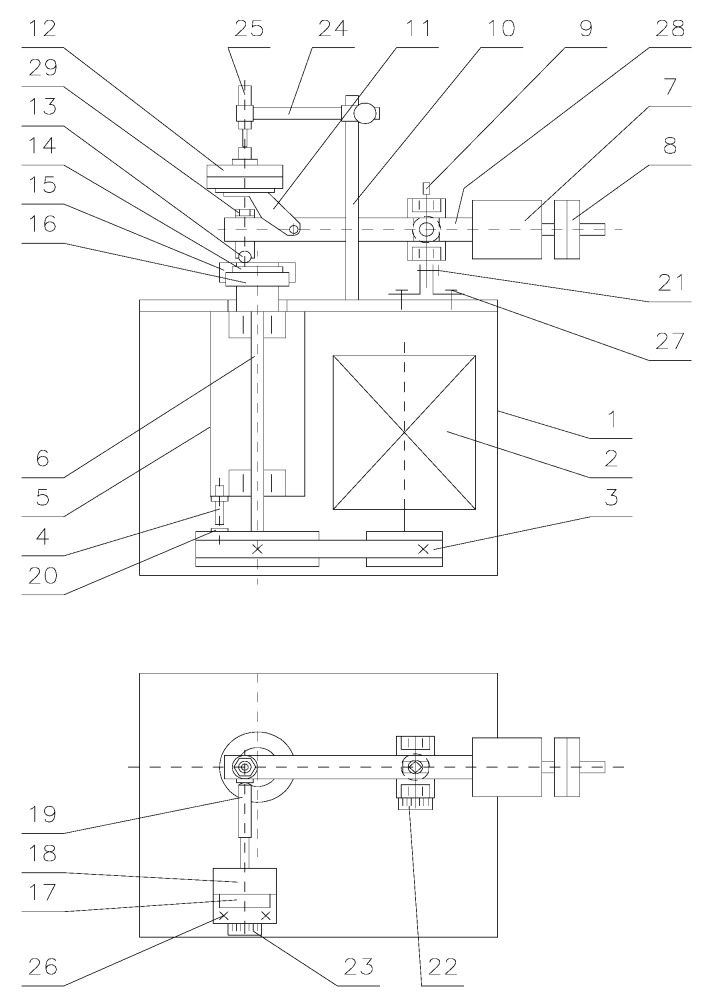
Schematic view of T-01M.

**Figure 4:**
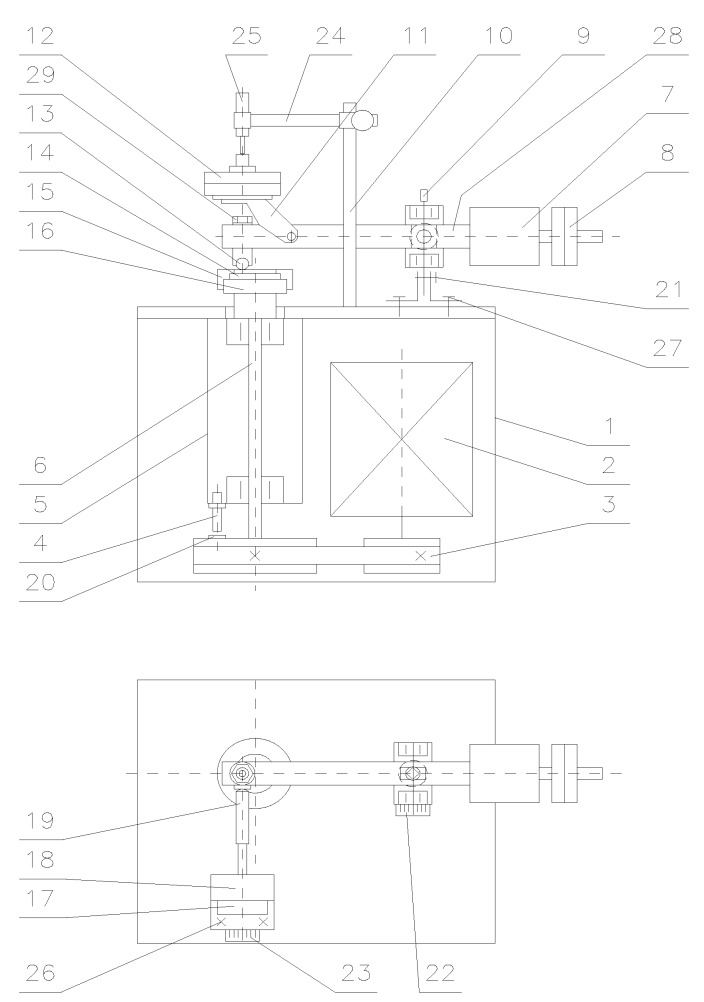
Kinematic scheme of the T-01M tribotester: 1—frame, 2—engine, 3—belt-drive gear, 4—impulse sensor (n revolution, SCID-1-ZVN from SENTRONIK SYSTEM, Stare Babice, Poland), 5—spindle sleeve, 6—spindle, 7—counterweight, 8—balancing weights, 9—rotational axis, 10—bracket, 11—weighing arm, 12—weights, 13—sample (pin/ball) with a temperature sensor (TP-202-K (K (NiCr-NiAl)) thermocouple from CZAKI Thermo-Product), 14—sample (disk), 15—disk clamping screw, 16—keep plate, 17—force sensor base, 18—friction force sensor (Hottinger’s tensometric force transducer type U1A (or S2)), 19—pusher, 20—n revolution sensor pin, 21—clamping screw, 22, 23—friction radius variation scale, 24—wear sensor arm, 25—inductive wear sensor (Hottinger’s W1T3 displacement probe), 26—force sensor base clamping screws, 27—rotational axis base, 28—friction pair loading arm, 29—sample clamping nut.

The tribological tests were performed in compliance with the ASTM G133 and G-99 standards using the T-01M tribotester produced by the Institute of Technology and Operation, Radom, Poland (Figure 3). The T-01M test stand consists of three basic units: the T-01M testing machine with BT-01 controller, the Spider8 digital amplifier (Darmstadt, Germany) and a computer system provided with specialist software for tribological testing (PC). A kinematic scheme of the T-01M with its sensor units is shown in Figure 4.

The basic sensor units of the T-01M tribotester include: a sensor unit for measuring friction force (tensometric friction force sensor), a sensor unit for measuring temperature in the vicinity of the friction pair, a sensor unit for measuring the spindle rotational speed, the number of revolutions and test duration (a system of impulse sensors), and a sensor unit for measuring wear (inductive wear sensor). The microprocessor control and measurement system (BT-01 controller) equipped with the Spider8 amplifier is used for making measurements (friction force, temperature in the vicinity of the friction pair, spindle rotational speed, the number of revolutions or test duration), engine control, and data archiving. Friction between the sample and the counter sample is measured by the tensometric force sensor unit. This unit consists of a tensometric force transducer (Hottinger’s tensometric force transducer type U1A (or S2), Darmstadt, Germany, measuring range: 50, 100 N, linearity deviation: 0.1) and the Spider8 amplifier.

**Figure 5:**
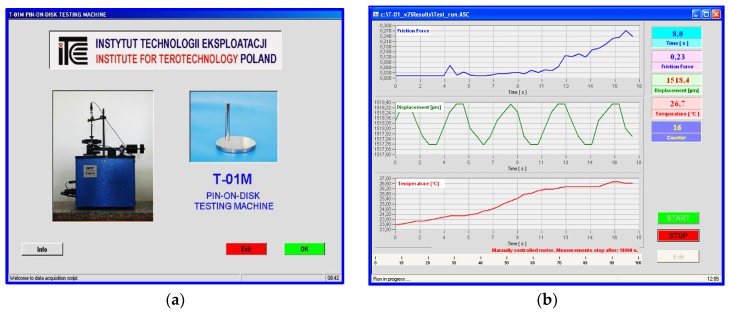
User interface of the T-01M pin-on-disk testing machine: (**a**) starting interface, (**b**) measurement interface.

Linear wear of the friction pair elements was measured using a system consisting of an inductive displacement transducer (Hottinger’s W1T3 displacement probe, Darmstadt, Germany; sensitivity tolerance: ± 1 mm, linearity deviation: 0.4, initial spring tension: 0.3 N) and the Spider8 amplifier. The displacement transducer unit was used for measuring the total linear wear of the friction pair elements (including the thermal expansion of friction pair elements) and for controlling disk alignment. Temperature measurements were made with the use of the TP-202-K (K (NiCr-NiAl)) thermocouple from the CZAKI Thermo-Product (Raszyn-Rybie, Poland) with the temperature range between –40 and 1000 °C and the accuracy of ±1 °C. The number of revolutions was measured with the use the SCID-1-ZVN impulse sensor from SENTRONIK SYSTEM with the measuring range of 1 mm. A user interface of the T-01M testing machine is shown in Figure 5.

**Figure 6:**
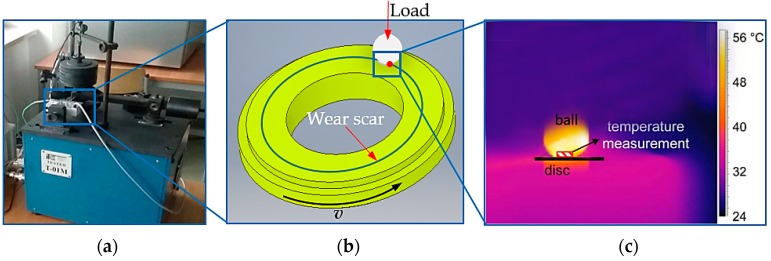
Tribological test stand: (**a**) T-01M, (**b**) ball-on-disk configuration, (**c**) thermal image from an additional independent temperature measurement captured with the FLIR X6580SC camera (Wilsonville, OR, USA).

Such devices are among the world’s most popular tribometers, and they are used for testing friction and wear of friction pairs in both concentrated contact (ball-disc) and distributed contact (pin-on-disc). The T-01M testing machine (Figure 6a) can be used for determining the following: frictional and wear characteristics of various combinations of materials and coatings; sliding properties of self-lubricating bearing materials and low-friction coatings; the effect of heat treatment and surface hardness of the tested material on its wear; the effect of surface layer treatment on its wear; friction surface wear; friction and anti-wear properties of cooling liquids. A unique application of the T-01M testing machine is that it can be used for testing combinations of polymeric materials (it allows electric insulation of the samples from the disk and measurement of the electrical resistance of metallic contacts, particularly when testing polymer coatings) that were fabricated by applying them onto the working surface of the disk or for testing samples of coatings that were applied to improve durability and sliding properties.

The T-01M tribotester allows testing the wear resistance and friction coefficients of any material combination working in a sliding motion, depending on the pressure force and sliding speed. These tribological tests were carried out using the ball-on-disc association, shown in Figure 6b. The rotational disc was made of samples made of polymer composites, while the stationary ball was made of aluminium oxide (Al_2_O_3_) counter-samples having 6 mm in diameter.

The tribological tests were carried out under dry friction conditions for different loading forces: 10N, 20N and 30N. The sliding speed was set according to the guidelines at 450 rpm and the test time was 1250 s. The friction radius was set equal to 17 mm in all tests. The test cycle consisted of 9367 full turns of the sample. The tests were carried out at ambient temperature. The parameters of the friction process are presented in Table 2. 

**Figure 7:**
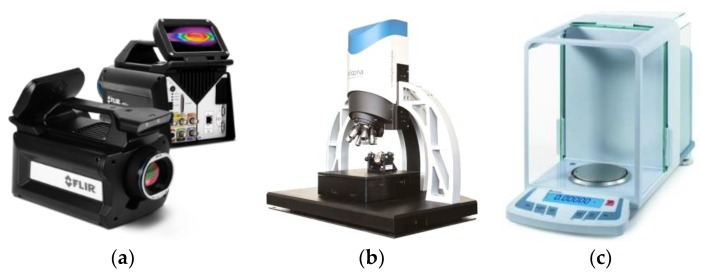
Additional equipment used in the tribological tests: (**a**) fast-speed thermal imaging camera FLIR X6580SC, (**b**) 3D optical measuring device InfiniteFocus G5 from Alicona, (**c**) Ohaus Discovery digital analytical scales for weighing the samples.

The tests involved measuring the temperature of the friction pair and the friction force to calculate the friction coefficient of the mating materials. The K (NiCr-NiAl) thermocouple was placed at a height of 2 mm from the friction surface, as shown in Figure 6. An additional independent (check) measurement was made using the state-of-the-art thermal imaging camera FLIR X6580SC for research purposes. The camera was provided with a cooled InSb infrared detector (Goettingen, Germany) allowing the capture of sharp images with 640 × 512 pixels in resolution (Figure 7a). 

**Figure 8:**
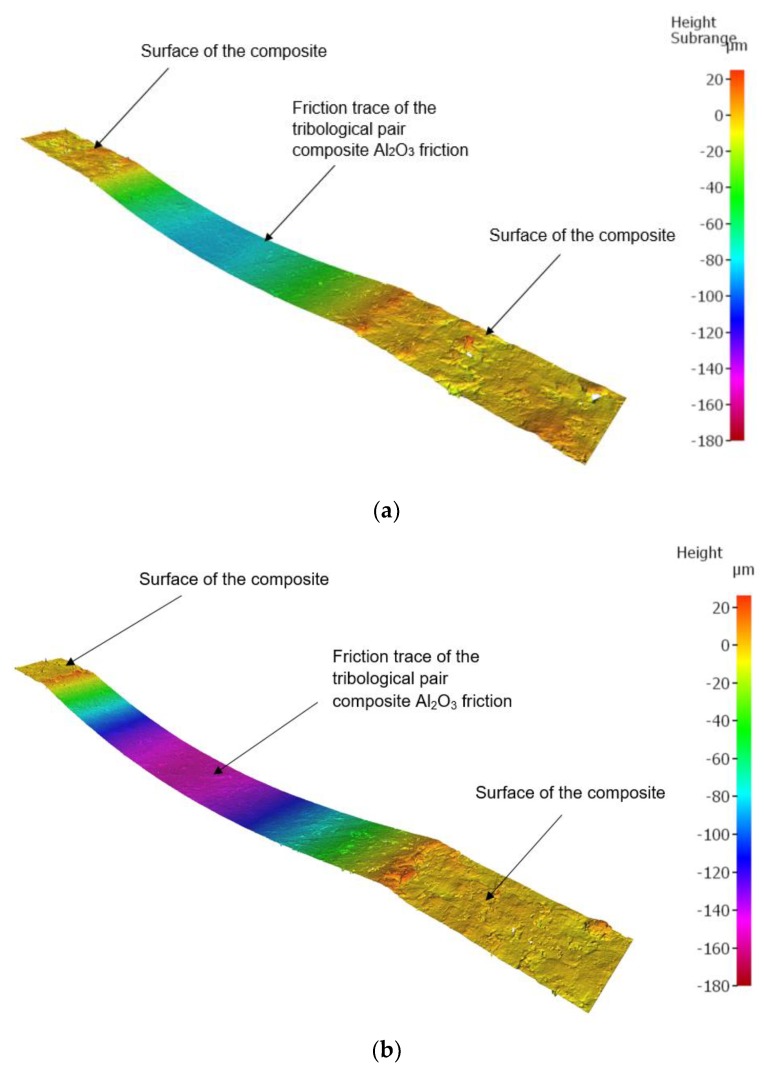
Image of the friction trace captured with the 3D optical measuring device InfiniteFocus G5 from Alicona for PTFE with graphite under a load of: (**a**) 20 N, (**b**) 30 N.

**Figure 9:**
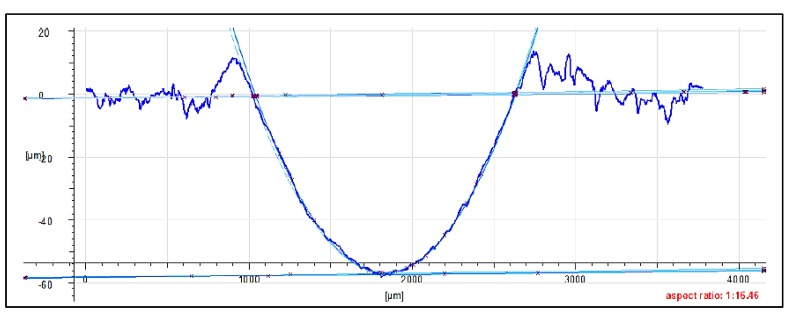
Friction trace of PTFE with graphite under a load of 30 N.

The friction traces were subjected to additional tests. Topography measurement and evaluation of the surface wear mechanism were performed with the use of 3D optical measuring device InfiniteFocus G5 (Raaba, Graz, Austria) from Alicona (Figure 7b). The images were obtained in a digital form and processed with the use of IT tools (Figure 8 and Figure 9). The results were used to calculate the friction trace surface, which made it possible to determine the wear volume of the sample. The wear volume was calculated as a product of the mean friction trace surface area and the friction trace diameter in the ball-on-disc test using Equation (1). The friction trace surface area (in the equation) was calculated using the profiles perpendicular to the friction path. A friction trace profile obtained for PTFE with graphite under a load of 30 N is shown in Figure 9:

(1) $$V_{f}=A\times L$$

where *V_f_* is the wear volume [mm^3^], *A* is the mean cross-sectional area of the friction trace [mm^2^], and *L* is the length of the stroke [mm].

To determine mass loss, the samples were weighed before and after the friction tests using the Ohaus Discovery digital analytical scales (Parsippany, NJ, USA) with a capacity of up to 210 g and a readability of ±0.01 mg (Figure 7c). Prior to weighing, the samples were cleaned with extraction naphtha and dried. The result is an average of five measurements. Based on the sample weight, it was possible to determine the mass loss *Z_m_* using the equation:

(2) $$Z_{m}=\frac{1}{5}\overset{5}{\underset{i=1}{\sum }}\left(m_{1i}-m_{2i}\right),$$

where *m*_1*i*_ is the sample mass before the friction test, *m*_2*i*_ is the sample mass after the friction test, and *i* is the number of test repeats. A comparison of the results is shown graphically in Section 3.

## 3. Results and Discussion

This section presents the results of tribological tests of polymeric composites (PA6 G with oil, PA6-G with MoS_2_, POM with aluminium, PET with PTFE, PTFE with bronze, PTFE with graphite) cooperating with aluminium oxide Al_2_O_3_ under sliding friction conditions, without lubricant and at ambient temperature.

Results of the experimental tests and calculations determined the characteristics of variation in selected tribological parameters as a function of time t. The temperature T of the friction node and the friction force Ft were measured in the tests. Based on obtained values of the friction force Ft, the coefficient of friction μ was determined too. The results demonstrate that all variations are nonlinear. They change dynamically as a function of time t. 

**Figure 10:**
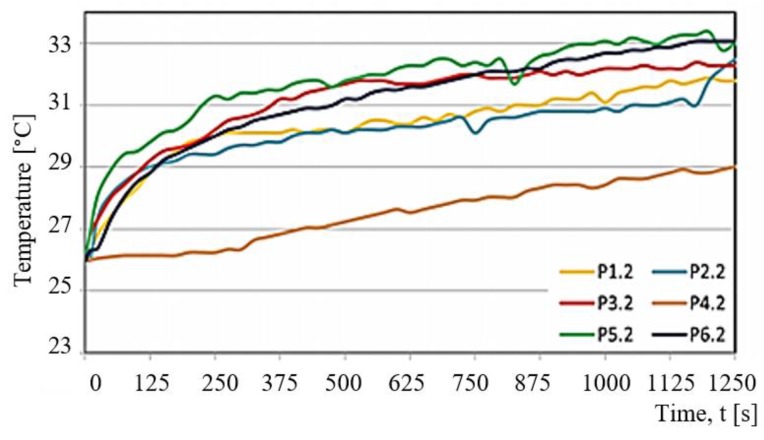
Temperature T of the friction pair as a function of time t, obtained at load P = 30 N for selected friction pairs Pij.

**Figure 11:**
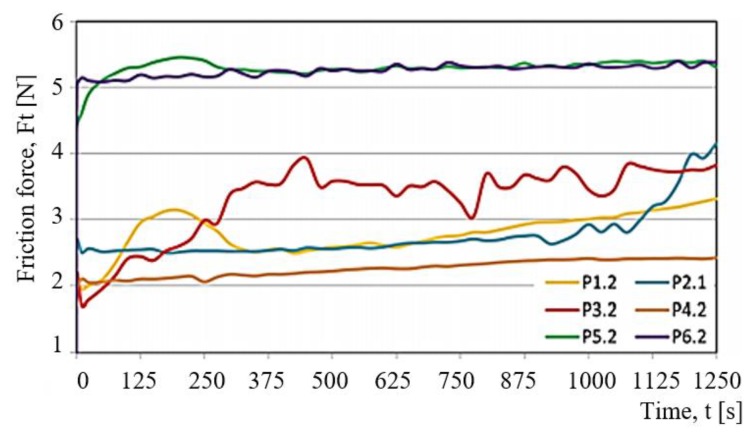
Variation in the friction force Ft as a function of time t, obtained at load P = 30 N for selected friction pairs Pi.j.

Figure 10 shows examples of changes in the temperature T of a friction node observed for selected test samples. The samples are denoted by Pi.j (P1.2, P2.2, P3.2, P4.2, P5.2, P6.2). Both the determination of the samples and the associated friction pairs are presented in Table 1 and Table 2. Figure 11 shows the changes in the friction force Ft as a function of time t for selected Pi.j samples loaded with P = 30 N.

For each of the samples, the friction pair temperature in the sliding process was not higher than 34 °C. Considering the temperature of the friction pair, the least favourable material was PTFE with bronze (P5.2). A characteristic feature of this friction pair is a sudden increase in the temperature, especially at the beginning of the sliding process. PET with PTFE (P4.2) has the most desired curve. It is characterised by a stable and low maximum temperature that does not exceed 29 °C. The changes in the temperature of the friction pair are almost linear during the entire test.

Changes in other tribological characteristics of the friction pairs (P1.1, P2.2, P3.2 and P6.2) are very similar, both in terms of the character and temperature of the friction pair. Apart from the P4.2 test, the temperature changes in the friction node can be considered as first order inertial segment. The curves shown in Figure 11 for the friction pairs P1.2, P2.2, P3.2, P4.2, P5.2, and P6.2 reveal differences in the nature of variation and high discrepancies between the friction force Ft. The lowest friction force was obtained for the P4.2 sample. The behaviour of the friction force Ft is close to linear. In short, it can be stated that there is a proportional relationship between the friction force Ft and the time of friction t. This change depends on the wear process in the friction pair and is almost linear.

The friction force Ft is the highest for the samples of P5.2 (PTFE with bronze) and P6.2 (PTFE with graphite). The force curve for both samples is almost identical to the curve showing the temperature T. Despite the fact that Ft of the two PTFE-based composites is about 5.2 N, it is very stable during the entire sliding period. The force Ft, however, achieves here much higher values than is the case with other tested samples.

**Figure 12:**
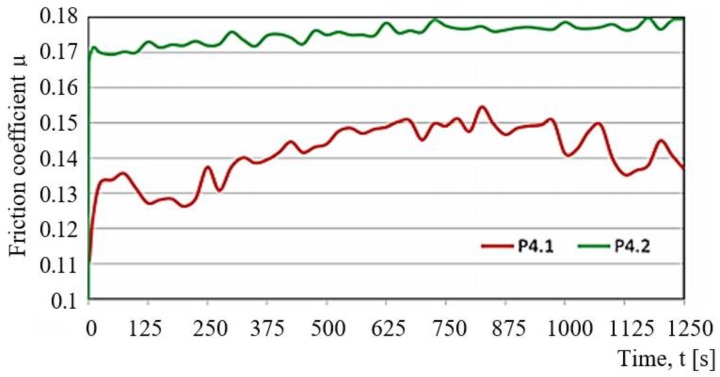
Variation in the friction coefficient as a function of time t, obtained at load P = 30 N (green) and P = 20 N (red).

**Figure 13:**
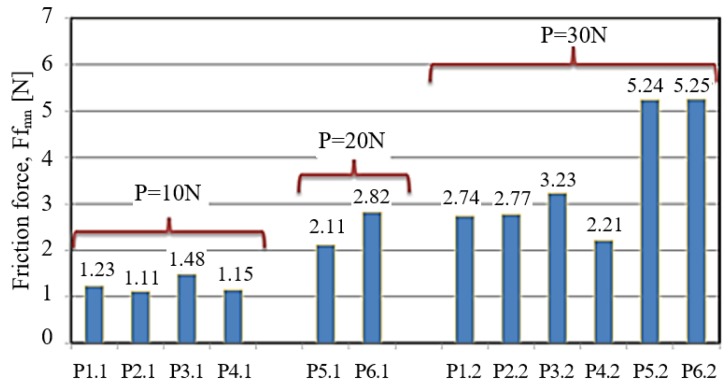
Comparison of the mean friction force Ff_mn_ for different load of the friction pair.

The samples of POM with aluminium (P3.2) are characterised by the most varied fluctuations in the friction force amounting to 1N (this behaviour occurs after 250 s of friction); the fluctuations are not cyclical. This curve is influenced by the accumulation of material at the contact point as well as by the dynamic change of friction conditions. The samples of PA6-G with MoS_2_ (P2.2) and oil (P1.2) show similar variations in the friction force Ft. The force is about 2.7 N. The only differences can be observed at the beginning and at the end of the sliding process. At the beginning of the sliding process, the friction force of PA6-G with oil is high. Next, it becomes stable and remains almost linear until the end of the process. An opposite trend can be observed for PA6-G with MoS_2_. In this case, the friction force increases in the final stage of the sliding process. The highest stability of the curve in the whole friction period is observed for the PET composite with PTFE (P4.2). In this case, the friction force does not exceed 2.5 N. Figure 12 shows the variations in the friction coefficient as a function of time t, obtained at the loads P = 30 N (green) and P = 20 N (red). Figure 13 shows the comparison of the friction force Ff_mn_* of every tested sample. *F = Force, f = friction, mn = mean.

Analysing Figure 13, it can be observed that the mean friction forces Ff_mn_ of the tested polymer composites differ from those obtained for PTFE with bronze (P5.1/P5.2) and PTFE with graphite (P6.1/P6.2). The friction forces of PTFE with bronze and PTFE with graphite are high during the test. This could mean that the friction pair has a higher resistance to motion and thus a greater amount of heat is generated. However, looking at the friction pair loads, this phenomenon cannot be unequivocally confirmed. Nevertheless, we can observe a proportional dependence between the friction force Ff_mn_ and the friction pair element loading. By increasing the loading force by 10 N for PTFE with bronze (P5.1/P5.2) and PTFE with graphite (P6.1/P6.2), Ff_mn_ increases in the range from 2.43 N to 3.13 N.

Figure 13 also shows an almost two-fold increase in the friction force parameters and a three-fold increase in the friction pair element loading for PA6-G with oil (P1.2/P1.2), PA6-G with MoS_2_ (P2.1/P2.2), POM with aluminium (P3.1/P3.2) and PET with PTFE (P4.1/P4.2). Considering PET with PTFE (P4.1/4.2) at a load of 20 N, it can be observed that the friction force Ff_mn_ increased by more than 1 N.

**Figure 14:**
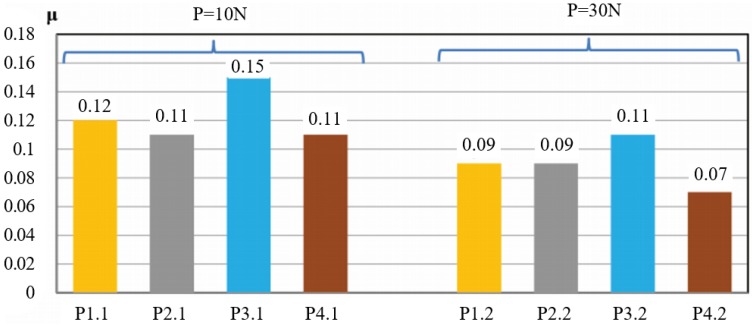
Variations in the mean coefficient of friction μ obtained for selected composites (P1.1/P1.2, P2.1/P2.2, P3.1/P3.2 P4.1/P4.2.

Figure 14 shows the changes in the mean coefficient of friction obtained for individual composites, depending on different values of the normal component loading of the friction pair (P = 10 N, P = 20 N).

**Figure 15:**
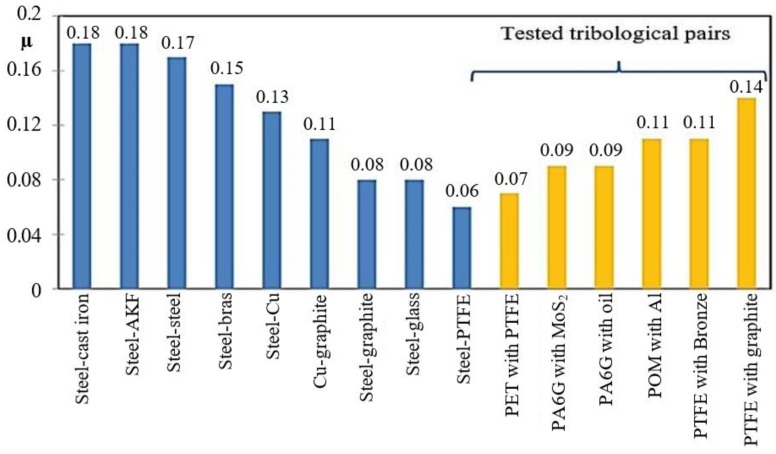
Comparison of the friction coefficients obtained for the tested composite materials and typical combinations of other structural materials.

A comparison of the results in Figure 13 does not take account of the PTFE-based composites because at P = 30 N their forces are much higher, as a result of which their coefficient of friction μ almost increased to 0.18. The composites loaded with 10 N have the approximate coefficient of friction ranging μ = 0.11–0.12. Only POM with aluminium has the coefficient of friction amounting to μ = 0.15. On increasing the load by 20 N, the coefficient of friction decreases (on average by 0.03) in all samples. Thus, the composites based on PA6-G did very well in the test. PET with PTFE (P4.1/P4.2) has the lowest coefficient of friction μ, ranging between 0.07 and 0.11. This is the best result. Taking into account its low coefficient of friction, very good temperature characteristics as a function of time in the sliding process, as well as very good properties, it can be stated that this composite has the best tribological properties out of all tested composites. A comparison of the friction coefficients obtained for the tested composites with the friction coefficients of other combinations of tribological pairs available in literature (Figure 15) confirms that the results are very good.

The coefficient of friction below 0.1 is a very good result, especially if we observe that the composites were loaded with a concentrated force exerted by the ball made of aluminium oxide Al_2_O_3_. It follows that the composites can carry high loads exerted by a much harder material. At the same time, they maintain very good tribological parameters for material combinations such as steel-glass or steel-graphite. As far as PET with PTFE is concerned, the value of μ = 0.07 is similar to that of the steel- PTFE combination, but this composite can be used much more widely than PTFE itself. 

**Figure 16:**
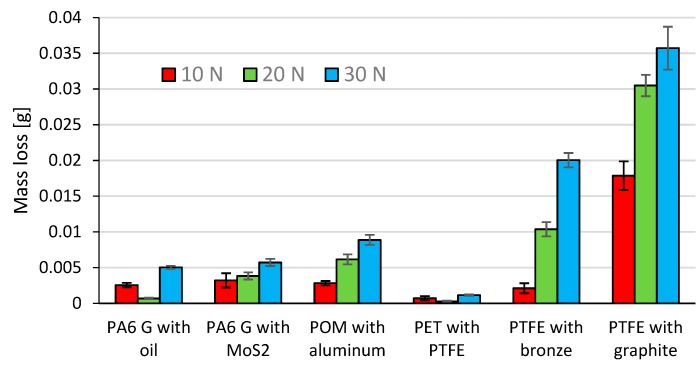
Mean mass loss of the samples.

Figure 16 shows the mean mass loss of the tested composite materials (samples) under the loads 10 N, 20 N, and 30 N. The mass loss of the counter samples (Al_2_O_3_ balls) is omitted due to insignificant values of friction traces. The data in Figure 16 reveal that the samples of PTFE with graphite and PTFE with bronze have the highest mean mass loss for every tested load (10 N, 20 N, 30 N). These composite materials also have a relatively higher coefficient of friction (µ ≈ 0.14) and lower hardness (60–65 Shore D, Table 1). The mean mass losses of PA6 G with oil and PA6 G with MoS_2_ are similar. The mass loss of POM with aluminium is slightly higher than those of PA6 G with oil and PA6 G with MoS_2_. The friction coefficients of the above composites are comparable (µ ≈ 0.1), which can be associated with the fact that their mass loss also results from their hardness ranging 83–85 Shore D (Table 1).

The lowest mean mass loss was obtained for PET with PTFE; this composite material is also characterised by the lowest friction coefficient (µ ≈ 0.07) and has the mean hardness of 70 Shore D. The results also demonstrate that, in the majority of cases, an increase in load leads to a higher mass loss of the composite materials. The exceptions were PA6 G with oil and PET with PTFE, for which the mass loss decreased under 20 N.

**Figure 17:**
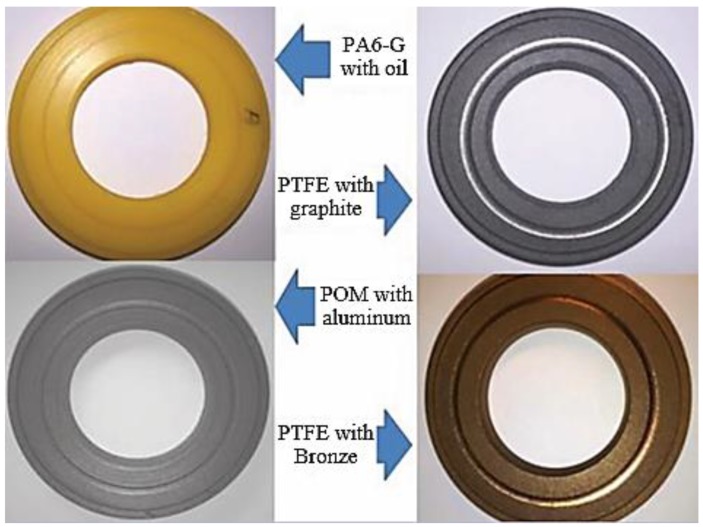
Test samples after the sliding process in a tribological test.

Figure 17 shows selected samples after the friction process. The visible friction traces result from contact between the composite materials and the Al_2_O_3_ ball. This can especially be observed for the samples of PTFE with graphite (P6.1, P6.2), as shown in Figure 17.

**Figure 18:**
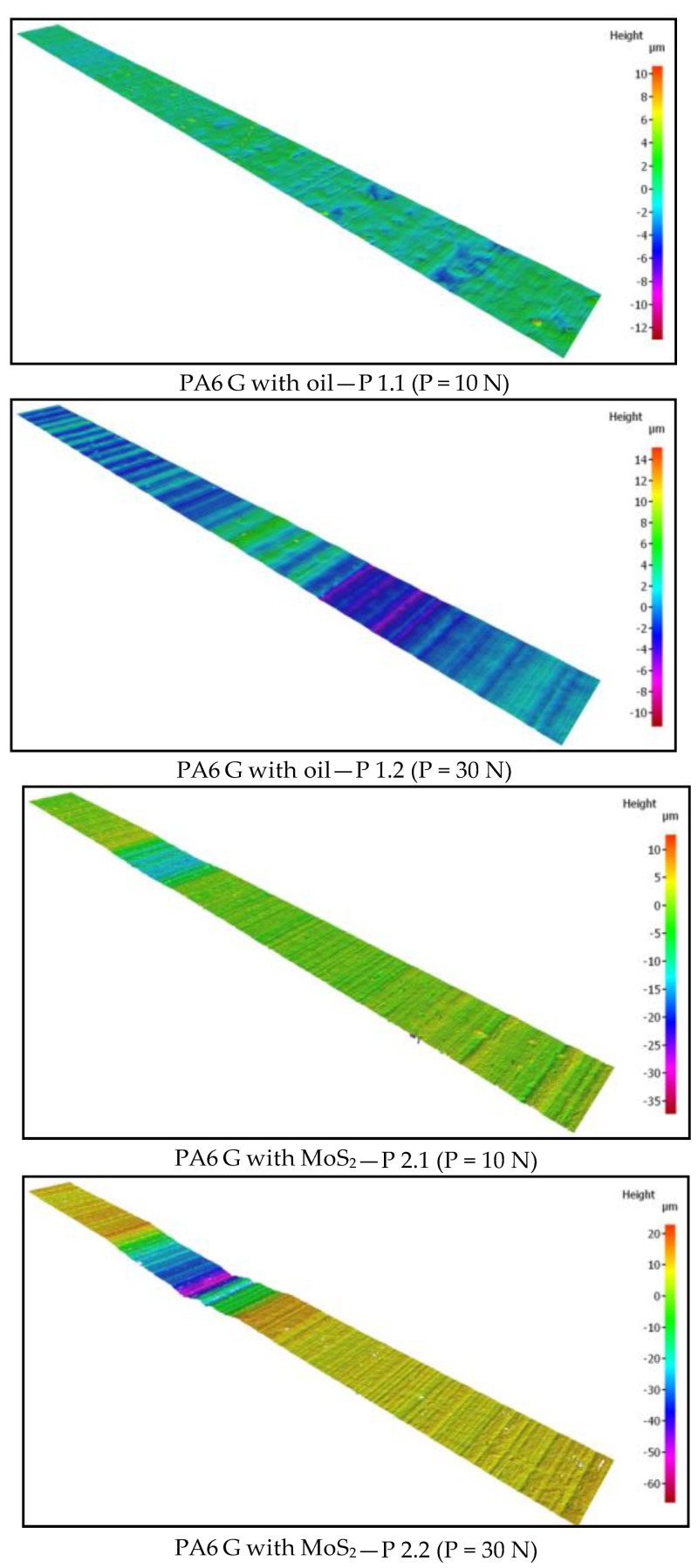

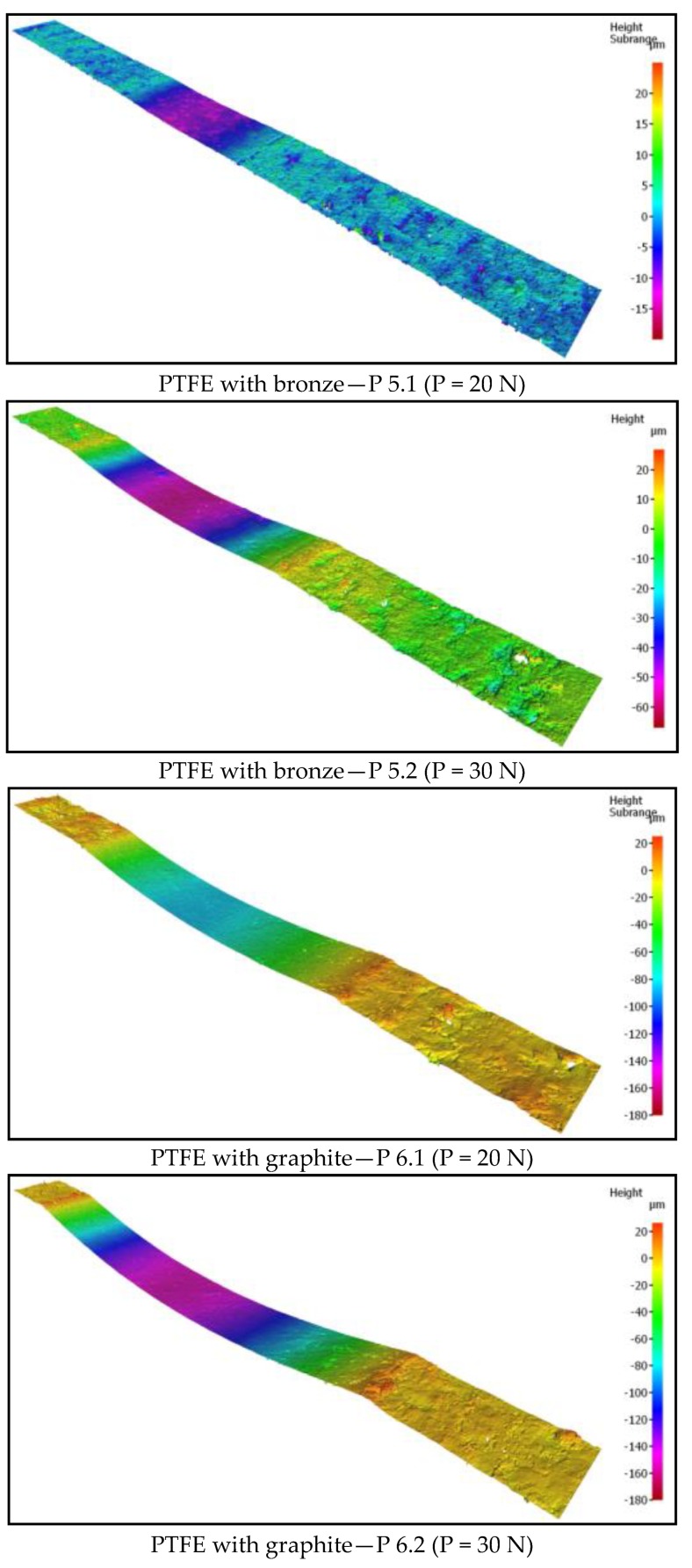
Selected microscopic images of the friction path of the sample under different loads: 10 N, 20 N, 30 N, captured with the InfiniteFocus G5 from Alicona.

Based on the obtained friction traces, it is possible to analyse phenomena occurring in the friction pair. As a result, it is possible to estimate the wear volume of a sample, among other things. The measurement of wear volume provides a more comprehensive assessment of wear than the measurement of linear wear. Knowing the wear volume of a sample, we can determine its approximate mass loss without checking its weight before and after friction testing. In effect, it is possible to determine mass loss (provided that the density of a composite material is known). Figure 18 shows selected microscopic images of the friction path of the tested sample under different loads: 10 N, 20 N, 30 N, captured with the InfiniteFocus G5 from Alicona. 

**Figure 19:**
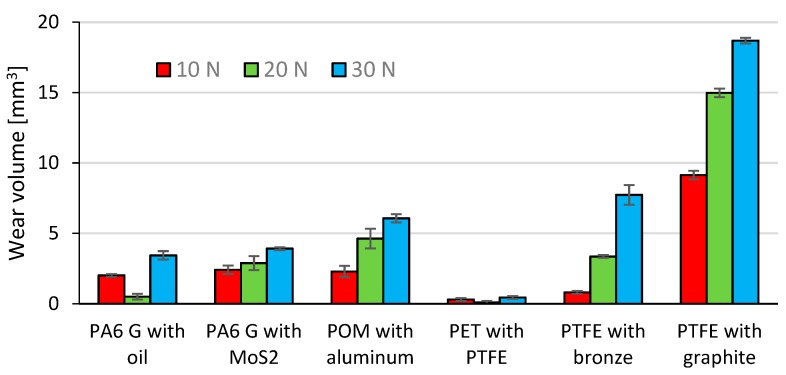
Wear volume of the tested composite materials.

Based on the characteristics shown in Figure 18, the wear volume was determined using the InfiniteFocus G5 from Alicona. The results of wear volume determined with Equation (1) are given in Figure 19. The diagrams in Figure 19 demonstrate that the highest mean wear volume was obtained for the samples of PTFE with graphite and PTFE with bronze under all tested loads (10 N, 20 N, 30 N). Similarly to the mass loss results, these composites have the highest wear volume. This results from their relatively high coefficient of friction (µ ≈ 0.14) and much lower hardness (60–65 Shore D, Table 1). It can also be observed that PA6 G with oil and PA6 G with MoS_2_ have similar mean wear volumes. 

The above composite materials have a similar coefficient of friction (µ ≈ 0.9) and similar hardness (83–85 Shore D; Table 1). The wear volume of POM with aluminium is slightly higher than that of PA6 G with oil and of PA6 G with MoS_2_.

The lowest mean wear volume was obtained for PET with PTFE. This material has the lowest coefficient of friction (µ ≈ 0.07), and its mean hardness is equal to 70 Shore D. The results also demonstrate that, in most cases, an increase in loading force leads to higher wear of the tested composite material. The exceptions were PA6 G with oil and PET with PTFE, for which the wear volume decreased under a load of 20 N. The results of wear volume and mass loss show a qualitative agreement. 

Figure 2 summarizes the roughness parameters Ra and Rsm measured on the samples surface after friction. The sample roughness obtained differed depending on the material. The highest values of the Ra parameter were for the PTFE with graphite sample and amounted to 1.95 µm. This material was also characterized by the largest parameter Rsm = 0.252 mm and the value of wear. The Rsm parameter defines the average width of profile elements. The smallest values of roughness parameters Ra were characteristic for POM with aluminum Ra = 0.57 µm and PET with PTFE where Ra = 0.74 µm. PET with PTFE material was also characterized by the lowest values of the parameter Rsm = 0.057 mm and wear. The obtained data indicate the impact of surface roughness parameters of the samples on the tribological properties of materials. Surface roughness can affect frictional resistance and intensify abrasive wear of materials.

**Figure 20:**
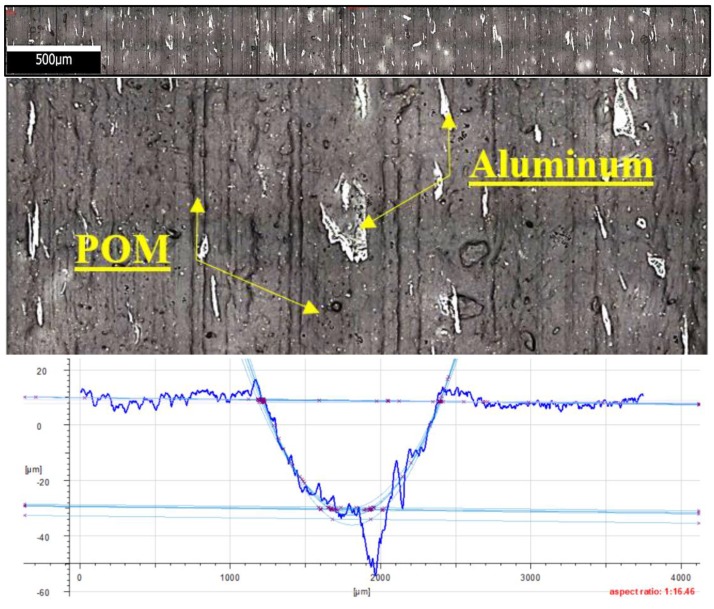
Microstructure and surface roughness profile after friction: POM with aluminium (×100).

**Figure 21:**
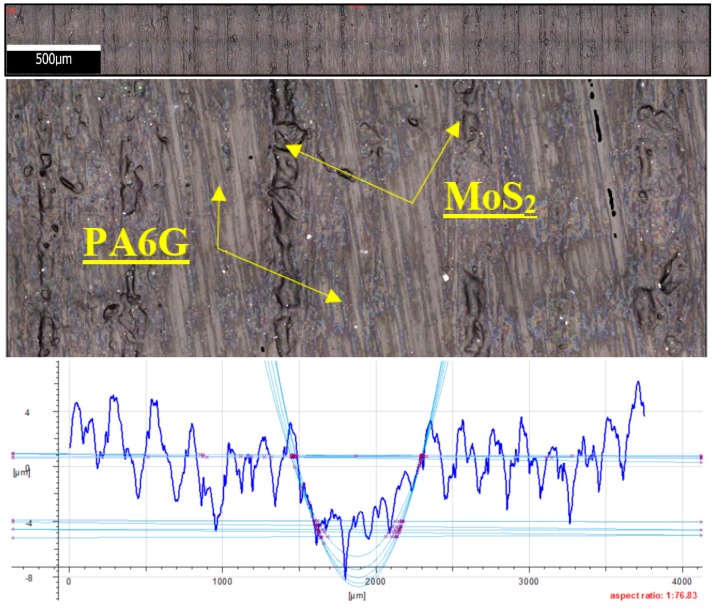
Microstructure and surface roughness profile after friction: PA6 G with MoS_2_ (×100).

Figure 20 shows the microstructure and surface roughness profile after friction of POM with aluminium (×100). The graphic images reveal the presence of a series of blurred, longitudinally distributed aluminium particles. The particles are distributed in parallel to the vector of linear velocity in friction. These microareas are characterized by intense friction and much higher temperatures when compared to other friction pairs. Figure 20 also shows that the aluminium particles are relatively evenly distributed in the POM matrix. The friction trace is visible in the plot, indicating a considerable degree of wear of the sample in the stabilized friction time. One can also notice characteristic deep grooves in the polymer matrix caused by the drifting aluminium particles. As a result, the friction surface properties, primarily hardness, are changed (aluminium versus polymer) along with the friction force and the coefficient of friction. This may also result from a different nature of surface roughness changes and sharper fluctuations in the friction force. The abrasive mechanism begins to dominate, which results in higher mass and volume consumption. Figure 21 shows the microstructure and surface roughness profile after friction of PA6 G with MoS_2_. As in the previous figure, the graphic images show that MoS_2_ particles are distributed lengthwise in the PA6 G matrix. The distribution of MoS_2_ particles is also parallel to the vector of linear velocity on the friction path. Although the accumulation of MoS_2_ particles in the friction trace is smaller, the particles are tight together, there is no sign of blurring between the longitudinal strips. The profilogram in Figure 21 also shows repeated mass loss of the PA6G matrix at the bottom of the friction path. 

**Figure 22:**
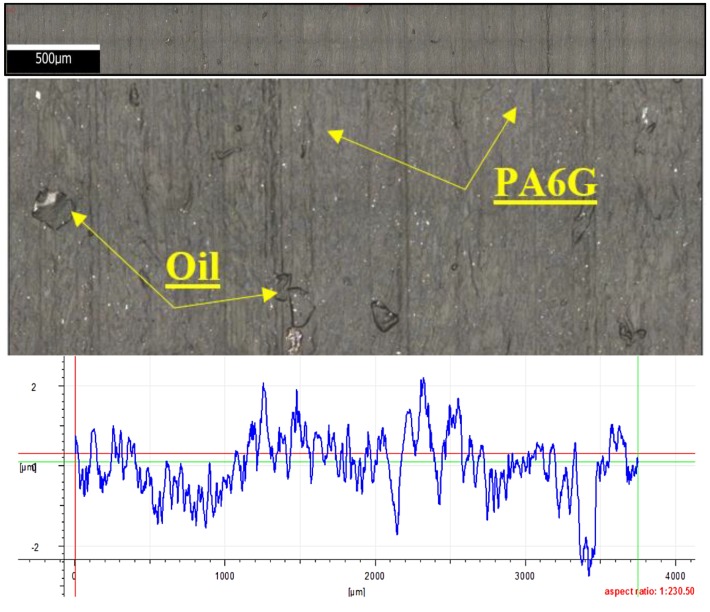
Microstructure and surface roughness profile after friction: PA6 G with oil (×100).

Figure 22 shows the microstructure and surface roughness profile after friction of PA6G with oil. The friction trace shows the presence of sphere-shaped oil particles in open pockets. These particles are either insignificant or invisible in the microscopic image of the friction path. The friction trace is insignificant and hard to identify, and this observation agrees with the profilogram obtained after measurement of the surface roughness profile after friction. Slight surface scratches are only visible at the bottom of the friction trace. During friction PA6 G with oil has better sliding effects, which results in a lower degree of wear. 

**Figure 23:**
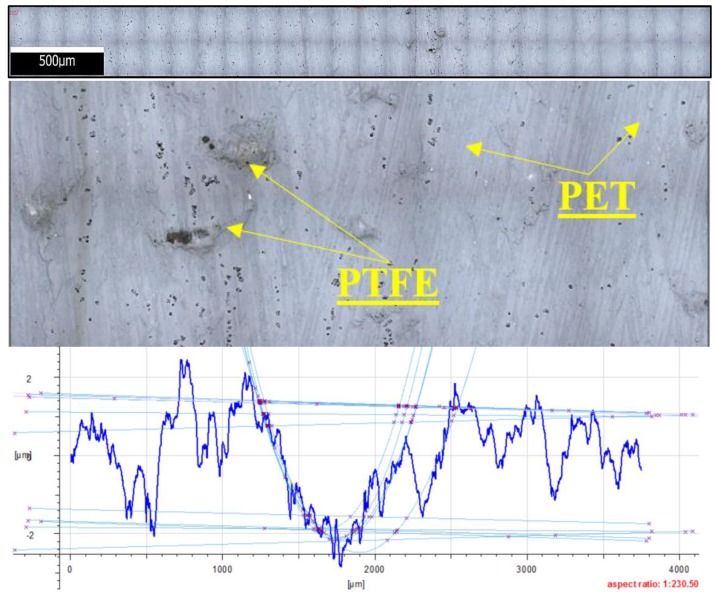
Microstructure and surface roughness profile after friction: PET with PTFE (×100).

Figure 23 shows the microstructure and surface roughness profile after friction of PET with PTFE. One can notice a characteristic accumulation of PTFE particles against the PET matrix, which results in considerable irregularities of the friction trace. This leads to the formation of microareas with heavy friction interactions, as a result of which the PTFE particles are pulled out and moved during friction, creating new clusters. This may also be caused by phenomena occurring on the PTFE and PA6G interphase. The phenomenon of pulling out PTFE particles from the matrix also causes changes in general friction conditions. The mechanism of abrasion becomes dominant here, which leads to higher mass loss and wear volume than in the case of other composite materials. 

**Figure 24:**
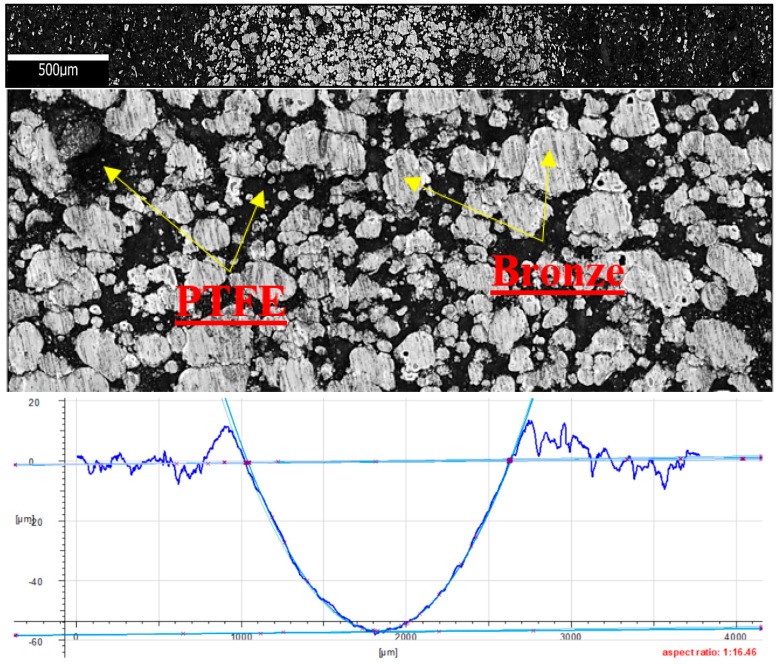
Microstructure and surface roughness profile after friction: PTFE with bronze (×50).

Figure 24 shows the microstructure and surface roughness profile after friction of PTFE with bronze. In the friction path’s centre, one can observe a significant increase in the filler (bronze) compared to the matrix (PTFE). The clusters of bronze particles are accumulated at the bottom of the friction path. The friction trace is regular on the surface, and a mass loss of the polymer matrix is practically invisible. The bronze particles are arranged regularly in the matrix, and their surface areas are similar. The profilogram reveals that the wear of PTFE with bronze is much higher than the wear measured for the samples of other tested composite materials. Figure 24 also reveals the presence of distinctive flash on the surface of the sample, at the end of the friction path. The flash is of a considerable size, reaching up to 12 mm. The graphic images given in Figure 24 indicate that abrasion is the dominant type of wear in this case. The fiction trace has a depth of up to 58 mm. 

**Figure 25:**
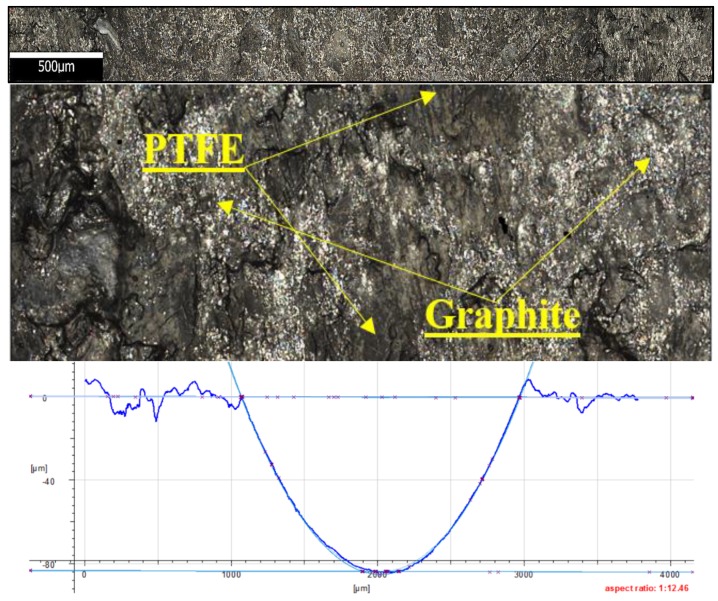
Microstructure and surface roughness profile after friction: PTFE with graphite (×50).

Figure 25 shows the microstructure and surface roughness profile after friction of PTFE with graphite. Due to the filler type (graphite), the profilogram does not show the characteristic flash in the upper part of the friction trace, as it was observed for PTFE with bronze in Figure 24. The depth of the friction trace is considerably high and is approx. 90mm. Considering the results, it is the highest wear value obtained for the tested composite materials. Another characteristic is the presence of graphite particles against PTFE. Brittle graphite is first pushed into the polymer and then lifted together with this material. The friction trace is regular and free from strips indicating mass loss. It can be observed that the friction trace is regular all over the circumference of the friction path.

## 4. Conclusions

The results of the study confirm that polymeric composites are an alternative to the existing sliding materials, especially metals. The advantage of using polymeric composites in sliding pairs is demonstrated by both the test results and their constructional reliability. In addition, this group of materials has great potential for development in the field of sliding materials used in friction pairs. This is facilitated by the possibility of their modification, which is basically a simpler solution than the search for a completely new sliding material. Eliminating internal structure defects with the use of appropriate additives is a much more cost-effective solution and does not require many additional tests. This cannot be said about developing completely new materials with similar tribological characteristics, which required conducting many different tests.

All tested polymeric composites with various additives are characterised by very good tribological properties, desirable when used in the construction of sliding elements. The set load values have an undeniable influence on the degree of wear. This is proven by the data presented in the tables and the characteristics shown in the diagrams. The PA6-G composite with mineral oil addition definitely has the best properties out of all tested composites. Despite their good results, the PTFE-based composites hold lower positions. This is due to their high price, which makes them cost-ineffective. 

In order to improve the sliding properties of friction pairs in devices and machines, all tested composites can be used as thin slip coatings or as replaceable elements (e.g., sleeves, bushings, bushings, inserts, etc.). If glass fibres are used, the stiffness, mechanical strength, and dimensional stability of these composite materials can be significantly improved. It should be noted that stiffness and stability are just as important as tribological properties of elements. They influence the characteristics of the linear displacement during friction. Thus, the curve obtained for the P1.1 sample is very unstable, while the PTFE composites with graphite and bronze show completely different characteristics. The study also showed that the optimal content of the filler, i.e., the additive in the polymer, is a very important factor influencing the results of tribological tests. Bronze, aluminium, graphite, and other fillers increase the temperature range of the polymer. Important conditions in the friction pair also change. PA6-G with oil is the best choice for thin sliding coatings out of all tested composites. On the other hand, if the whole sliding pair element is to be made of a composite material, it is more recommended to use PTFE with graphite or bronze. The results demonstrate that, in most cases, an increase in loading force leads to a decrease in the coefficient of friction of the tested composite materials. This results from an increased content of the filler (oil, bronze, graphite, etc.), which significantly improves the friction conditions. The highest mean mass loss and wear volume were obtained for the samples of PTFE with graphite and PTFE with bronze for every tested loading force (10 N, 20 N, 30 N). This can be explained by the fact that—compared to other composites—these composite materials have a relatively higher coefficient of friction (µ ≈ 0.14) and much lower hardness (60–65 Shore D, Table 1). PA6 G with oil and PA6 G with MoS2 have similar mean mass loss and wear volume. These composite materials have similar coefficients of friction (µ ≈ 0.9) and hardness (83–85 Shore D; Table 1). The mass loss and wear volume of POM with aluminium is slightly higher than that obtained for PA6 G with oil and PA6 G with MoS_2_. The results of this study have shown that the lowest mean wear volume was obtained for PET with PTFE; this composite material has the lowest coefficient of friction (µ ≈ 0.07) and its mean hardness is 70 Shore D. The results also demonstrate that, in the majority of cases, an increase in loading force leads to higher wear of the tested composite material. The only exceptions were PA6 G with oil and PET with PTFE, for which the wear volume decreased under a load of 20N. The results of mass loss and wear volume show a qualitative agreement. The obtained microstructures and surface roughness profiles after friction, shown in Figure 20, Figure 21, Figure 22, Figure 23, Figure 24 and Figure 25, confirm the results of tribological measurements of the analysed composite materials. 

## Figures and Tables

**Table 1:** Comparison of tested polymer composites.

Characteristics	Type of Polymeric Composite					
	PA6 G with Oil	PA6 G with MoS_2_	POM with Aluminium	PET with PTFE	PTFE with Bronze	PTFE with Graphite
Density [g/cm³]	1.14	1.15	1.38	1.6	3.8	2.15
Content of the additive [%]	15 (10–30)	20 (10–30)	20 (15–35)	20 (10–20)	25 (10–45)	25 (15–30)
Symbol	P1.1/P1.2	P2.1/ P2.2	P3.1/ P3.2	P4.1/ P4.2	P5.1/ P5.2	P6.1/P6.2
Long-term operating temperature range [°C]	−40–100	−30–100	−30–105	−30–160	−200–220	−200–220
Thermal conductivity [W/K*m]	0.23	0.23	no data	0.30	0.77	0.93
Modulus of elasticity [GPa]	3.1	3.2	no data	3.2	no data	0.72
Shore D hardness, 15s—value	85	83	no data	70	65	60
Ball indentation hardness, H_358/30_ [MPa]	140	150	no data	183	40	32
Melting temperature [°C]	220	220	no data	249	320	320

**Table 2:** Friction process data.

List of Parameters of the Friction Process						
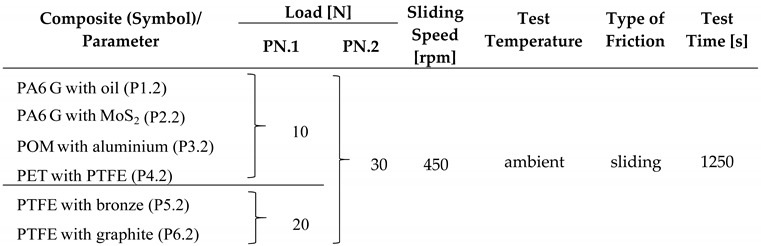						

## References

[1] Burakowski T., Wierzchon T. (1998). Surface Engineering of Metals: Principles, Equipment, Technologies, Materials Science & Technology.

[2] Söffker D., Rothe S. (2017). New Approaches for Supervision of Systems with Sliding Wear: Fundamental Problems and Experimental Results Using Different Approaches. Appl. Sci..

[3] Dziedzic K., Pashechko M., Barszcz M., Józwik J. (2017). Structure and Construction Assessment of the Surface Layer of Hardfaced Coating after Friction. Adv. Sci. Technol. Res. J..

[4] Gualco A., Svoboda H.G., Surian E.S. (2016). Study of abrasive wear resistance of Fe-based nanostructured hardfacing. Wear.

[5] Chen B., Wang J., Yan F. (2011). Friction and Wear Behaviors of Several Polymers Sliding Against GCr15 and 316 Steel Under the Lubrication of Sea Water. Tribol. Lett..

[6] Waddad Y., Magnier V., Dufrénoy P., De Saxc G. (2019). Multiscale thermomechanical modeling of frictional contact problemsconsidering wear–Application to a pin-on-disc system. Wear.

[7] Pashechko M.I., Montusiewicz J. (2012). Evaluation of the Wear Resistance of Eutectic Coatings of the Fe–Mn–C–B System Alloyed by Si, Ni, and Cr Using Multi-Criteria Analysis. Mater. Sci..

[8] Pashechko M., Montusiewicz J., Dziedzic K., Jozwik J. (2017). Multicriterion Assessment of Wear Resistance of Fe–Mn–C–B Eutectic Coatings Alloyed with Si, Ni, and Cr. Powder Met. Met. Ceram..

[9] Khonsari M.M., Booser E.R. (2017). Applied Tribology: Bearing Design and Lubrication.

[10] Sudeepan J., Kumar K., Barman T., Sahoo P. (2014). Study of Tribological Behavior of ABS/ CaCO_3_ Composite Using Grey Relational Analysis. Procedia Mater. Sci..

[11] Fox-Rabinovich G.S., Totten G.E. (2006). Self-Organization during Friction, Advance Surface Engineered Materials and Systems Design.

[12] Shahroozi A., Afsari A., Khakan B. (2018). Microstructure and mechanical properties investigation of Stellite 6 and Stellite 6/TiC coating on ASTM A105 steel produced by TIG welding process. Surf. Coat. Technol..

[13] Siemiątkowski Z., Szumiata T., Gzik-Szumiata M., Martynowski R., Rucki M. (2018). Application of the microscopic and Mössbauer studies to the analysis of a marine diesel engine crankshaft. J. Mar. Eng. Technol..

[14] Corrêa E.O., Alcântara N., Valeriano L., Barbedo N., Chaves R. (2015). The effect of microstructure on abrasive wear of a Fe–Cr–C–Nb hardfacing alloy deposited by the open arc welding process. Surf. Coat. Technol..

[15] Paszeczko M., Dziedzic K., Mendyk E., Jozwik J. (2018). Chemical and phase composition of the friction surfaces Fe-Mn-C-B-Si-Ni-Cr hardfacing coatings. J. Tribol..

[16] Borba N., Blaga L., Dos Santos J., Amancio-Filho S. (2018). Direct-Friction Riveting of polymer composite laminates for aircraft applications. Mater. Lett..

[17] Panda A., Dyadyura K., Valíček J., Harničárová M., Zajac J., Modrák V., Pandová I., Vrábel P., Nováková-Marcinčínová E., Pavelek Z. (2017). Manufacturing Technology of Composite Materials—Principles of Modification of Polymer Composite Materials Technology Based on Polytetrafluoroethylene. Materials.

[18] Dahy H. (2019). Natural Fibre-Reinforced Polymer Composites (NFRP) Fabricated from Lignocellulosic Fibres for Future Sustainable Architectural Applications, Case Studies: Segmented-Shell Construction, Acoustic Panels, and Furniture. Sensors.

[19] Friedrich K. (2018). Polymer composites for tribological applications. Adv. Ind. Eng. Polym. Res..

[20] Kurdi A., Chang L. (2019). Recent Advances in High Performance Polymers—Tribological Aspects. Lubricants.

[21] Agrawal S., Singh K., Sarkar P. (2018). Comparative investigation on the wear and friction behaviors of carbon fiber reinforced polymer composites under dry sliding, oil lubrication and inert gas environment. Mater. Today Proc..

[22] Prabhakar K., Debnath S., Ganesan R., Palanikumar K. (2018). A review of mechanical and tribological behaviour of polymer composite materials. IOP Conf. Ser. Mater. Sci. Eng..

[23] Gevorkyan E., Lavrynenko S., Rucki M., Siemiatkowski Z., Kislitsa M. Preparation of nanostructured materials by electrical sintering. Proceedings of the 7th International Conference on Mechanics and Materials in Design.

[24] Brostow W., Kovačević V., Vrsaljko D., Whitworth J. (2010). Tribology of polymers and polymer-based composites. J. Mater. Educ..

[25] Myshkina N.K., Pesetskiia S.S., Grigorieva A.Y. (2015). Polymer Tribology: Current State and Applications. Tribol. Ind..

[26] Kim J., Jang H. (2014). Friction and wear of monolithic and glass-fiber reinforced PA66 in humid conditions. Wear.

[27] Skoneczny W., Kaptacz S., Barylski A., Kmita T. (2018). Analysis of tribological properties of selected ptfe-based polymer composites in a sliding interaction with aluminium oxide (Al_2_O_3_). Tribologia.

[28] Song J., Yu Y., Zhao G., Qiu J., Ding Q. (2019). Comparative study of tribological properties of insulated and conductive polyimide composites. Friction.

[29] Pogačnik A., Kupec A., Kalin M. (2017). Tribological properties of polyamide (PA6) in self-mated contacts and against steel as a stationary and moving body. Wear.

[30] Gebretsadik D.W., Hardell J., Prakash B. (2020). Friction and wear characteristics of PA 66 polymer composite/316L stainless steel tribopair in aqueous solution with different salt levels. Tribol. Int..

[31] Mao K., Greenwood D., Ramakrishnan R., Goodship V., Shrouti C., Chetwynd D., Langlois P. (2019). The wear resistance improvement of fibre reinforced polymer composite gears. Wear.

[32] Jozwik J., Dziedzic K., Paszeczko M., Barszcz M. Comparative Assessment of Tribological Properties of Selected Polymers and Polymer Composites. Proceedings of the 2019 IEEE 6th International Workshop on Metrology for AeroSpace (MetroAeroSpace).

[33] Siemiatkowski Z., Gzik-Szumiata M., Szumiata T., Rucki M., Martynowski R. (2017). Metallurgical quality evaluation of the wind turbine main shaft ^42^CrMo_4_ steel: Microscopic and Mssbauer studies. Nukleonika.

[34] Pecora A., Maiolo L., Minotti A., De Francesco R., De Francesco E., Leccese F., Cagnetti M., Ferrone A. Strain gauge sensors based on thermoplastic nanocomposite for monitoring inflatable structures. Proceedings of the 2014 IEEE Metrology for Aerospace (MetroAeroSpace).

[35] Siemiatkowski Z., Rucki M., Lavrynenko S. Investigations of the shrink-fitted joints in assembled crankshafts. Proceedings of the 7th International Conference on Mechanics and Materials in Design.

[36] Petritoli E., Leccese F., Leccisi M. Inertial Navigation Systems for UAV: Uncertainty and Error Measurements. Proceedings of the 2019 IEEE 5th International Workshop on Metrology for AeroSpace (MetroAeroSpace).

[37] Petritoli E., Leccese F. (2018). High Accuracy Attitude and Navigation System for an Autonomous Underwater Vehicle (AUV). Acta IMEKO.

[38] Petritoli E., Leccese F., Cagnetti M. A high accuracy buoyancy system control for an underwater glider (2019). Proceedings of the 2018 IEEE International Workshop on Metrology for the Sea, Learning to Measure Sea Health Parameters (MetroSea).

[39] Jozwik J. Dynamic Measurement of Spindle Errors of CNC Machine Tools by Capacitive Sensors during Aircraft Parts Machining. Proceedings of the 2018 5th IEEE International Workshop on Metrology for AeroSpace (MetroAeroSpace).

[40] Jozwik J. Measuring of Axis Errors and Their Prognosis during Aircraft Parts Machining. Proceedings of the 2018 5th IEEE International Workshop on Metrology for AeroSpace (MetroAeroSpace).

[41] Józwik J., Wac-Włodarczyk A., Michałowska J., Kłoczko E.M. (2018). Monitoring of the Noise Emitted by Machine Tools in Industrial Conditions. J. Ecol. Eng..

[42] Łukaszewicz A. (2018). Nonlinear Numerical Model of Friction Heating during Rotary Friction Welding. J. Frict. Wear.

[43] Grodzki W., Łukaszewicz A. (2015). Design and manufacture of unmanned aerial vehicles (UAV) wing structure using composite materials. Mater. Werkst..

[44] Sidun P., Łukaszewicz A. (2017). Verification of Ram-Press Pipe Bending Process using Elasto-Plastic FEM Model. Acta Mech. Autom..

